# Exploration of Carotenoid-Producing Microorganisms from the Kuril-Kamchatka Trench and Their Antioxidant Potential

**DOI:** 10.3390/md24030105

**Published:** 2026-03-11

**Authors:** Guan-Yuan Zhang, Xue-Gong Li, Hai-Rong Fang, Jin-Wei Gao, Wei-Jia Zhang

**Affiliations:** 1 Laboratory of Deep-Sea Microbial Cell Biology, Institute of Deep-Sea Science and Engineering, Chinese Academy of Sciences, Sanya 572000, China; 2 University of Chinese Academy of Sciences, Beijing 101408, China; 3 Institution of Deep-Sea Life Sciences, IDSSE-BGI, Sanya 572000, China; 4 Laboratory of Marine Geophysics and Georesources, Institute of Deep-Sea Science and Engineering, Chinese Academy of Sciences, Sanya 572000, China

**Keywords:** hadal zone, Kuril-Kamchatka Trench (KKT), carotenoid-producing microorganisms (CPMs), microbial diversity, antioxidant activity

## Abstract

Despite its extreme conditions, the hadal environment harbors abundant but largely underexplored microbial resources. In this study, samples from the Kuril-Kamchatka Trench (KKT) were enriched at low temperature using R2A and 2216E media. Carotenoid-producing microorganisms (CPMs) were isolated from approximately one-third of the samples, yielding a total of 124 isolated strains spanning 4 phyla and 11 genera. Planococcus, Kocuria, Paracoccus, and Exiguobacterium collectively accounted for 75.8% of the isolates. The choice of culture medium significantly influenced CPM diversity at the family and genus levels, though not at the phylum or class level. Water depth, sample type, and sediment layer also significantly affected CPM community structure. Carotenoid spectral profiles correlated with phylogenetic lineage, and closer phylogenetic relationships corresponded to greater similarity in carotenoid biosynthesis gene clusters. Antioxidant assays (FRAP, ABTS, DPPH) demonstrated strong total antioxidant and radical scavenging capacities in carotenoid extracts from Citricoccus, Kocuria, Arthrobacter, and Olleya. Scavenging activity toward ABTS or DPPH radicals varied significantly among genera, suggesting genus-specific antioxidant mechanisms. These findings highlight the hadal zone as a promising reservoir of diverse CPMs and a valuable source of novel carotenoids and antioxidant-producing strains with potential biotechnological applications.

## 1. Introduction

The hadal zone encompasses marine environments at depths exceeding 6000 m [1]. Characterized by high hydrostatic pressure (HHP), near-freezing temperatures, and perpetual darkness, it represents one of the most extreme habitats on Earth [2]. Despite limited input of predominantly recalcitrant organic matter from surface waters [3,4], this environment sustains active microbial communities that play crucial roles in deep-sea organic carbon deposition and transformation [4,5,6,7]. These distinctive physicochemical parameters of the hadal zone shape unique microbial assemblages and metabolic profiles that differ substantially from those found in shallower marine environments [1,8,9], positioning this zone as a natural reservoir of microbial diversity. Furthermore, through prolonged adaptation to such extreme conditions, hadal microorganisms may have evolved specialized metabolic pathways capable of biosynthesizing novel secondary metabolites with promising biotechnological potential [10].

The extreme HHP and low temperature in the hadal environment fundamentally challenge microbial physiology by disrupting processes such as DNA replication, transcription, and protein synthesis [11,12,13,14]. Under HHP conditions, the fluidity of the cell membrane decreases while its rigidity increases, leading to reduced transmembrane permeability of water and other small molecules, thereby affecting membrane-dependent transport and metabolic functions [15]. Simultaneously, HHP can induce an increase in intracellular reactive oxygen species (ROS) levels [16]. Accumulated ROS can attack iron-sulfur clusters [17] and sulfur-containing amino acid residues in proteins [18], causing structural alterations and functional loss; they can also damage DNA, disrupting nucleic acid integrity [19] and trigger lipid peroxidation [20], impairing cell membrane structure and thereby interfering with membrane-based biochemical processes [21].

Carotenoids, a class of lipid-soluble pigments with highly unsaturated conjugated double-bond structures, can embed into the cell membrane and mitigate the adverse effects of HHP by enhancing membrane fluidity [22]. For instance, studies on *Staphylococcus xylosus* have shown that under low-temperature stress, increased intracellular carotenoid content helps maintain normal membrane fluidity, whereas inhibition of carotenoid synthesis leads to a significant decline in membrane fluidity under low-temperature conditions [23,24]. Additionally, carotenoids possess strong antioxidant capabilities, effectively scavenging free radicals and reducing intracellular ROS levels, thereby protecting key biomolecules such as proteins and nucleic acids from oxidative damage [25].

Given these documented physiological roles, we hypothesize that carotenoid-producing microorganisms (CPMs) are particularly advantaged in the hadal zone, making it an ideal environment for discovering novel CPMs and their bioactive compounds. To test this hypothesis, we collected sediment and near-bottom seawater samples from the Kuril-Kamchatka Trench (KKT; 5951–9588 m depth). We employed low-temperature enrichment with R2A and 2216E media to isolate pigmented strains, leading to the successful cultivation of 124 CPMs. We further characterized these isolates by analyzing the spectral profiles and biosynthetic gene clusters of their carotenoids, and systematically evaluated their antioxidant and radical scavenging activities. The CPMs and active metabolites discovered in this study establish a critical resource foundation for exploiting microbial potential from deep-sea extreme environments.

## 2. Results

### 2.1. Sampling and Enrichment of Microorganisms from the KKT

**Figure 1:**
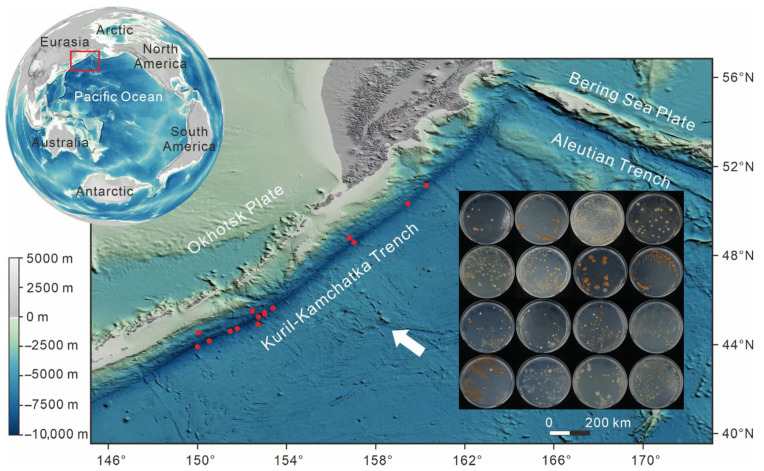
Overview of sampling stations in the Kuril-Kamchatka Trench during cruise TS42-1 and the pigmented colonies enriched therefrom. The red rectangle delineates the study area, solid red dots indicate sampling stations, and the inset displays representative enrichment culture plates of pigment-producing microorganisms.

During the TS42-1 expedition from June to July 2024, scientific investigations were conducted in the Kuril-Kamchatka Trench using the “Fendouzhe” manned submersible. Core sediment samples were collected from 15 stations along the trench axis via a mechanical arm (Figure 1). Simultaneously, near-bottom seawater was filtered on-site using the in-situ filtration system mounted on the submersible’s tail to obtain microbial biomass (approximately 200 L per station, with a filter membrane pore size of 0.22 μm). The sampling depth ranged from 5951 to 9588 m. After recovery to the deck, the sediment samples were diluted with sterile seawater, and the microorganisms from the filter membrane samples were suspended. Subsequently, aliquots of the suspensions were spread onto 2216E and R2A solid media plates and incubated at 4 °C in the dark. After approximately one month of incubation, microbial growth was observed on 510 plates, of which 170 plates contained pigment-producing colonies, accounting for about one-third. Notably, colony pigmentation were predominantly yellow, orange, and brown, some plates showed colonies of a single color, while others exhibited a mixture of multiple colors. Additionally, on some plates, pigmented colonies occupied an extremely high proportion, even covering the entire plate surface (Figure 1). These initial culturing results indicate that the deep environment of the Kuril-Kamchatka Trench harbors abundant pigment-producing microorganisms.

### 2.2. Diversity of Carotenoid-Producing Microorganisms from KKT

**Figure 2:**
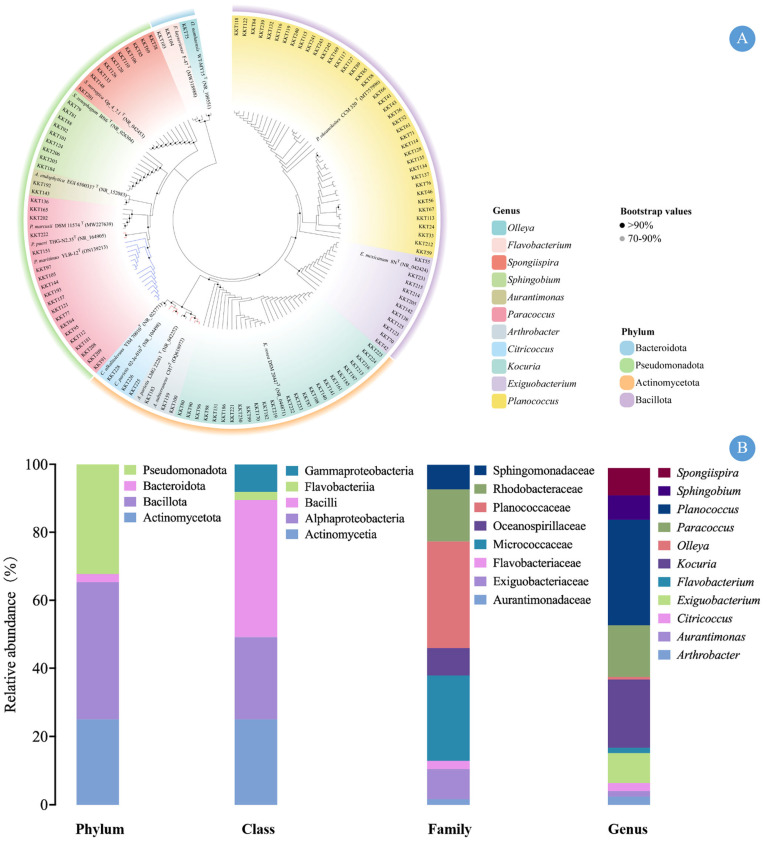
Phylogenetic tree and abundance of carotenoid-producing microorganisms: (**A**) Phylogenetic tree of CPMs isolated from KKT and their closest relatives from GenBank, constructed based on 16S rRNA gene sequences using the neighbor-joining method. The 16S rRNA sequences are aligned with the CLUSTAL W program in MEGA 12, and evolutionary distances are computed using Kimura’s two-parameter model. Bootstrap values greater than 70% based on 1000 replicates are shown at the nodes. (**B**) Community structure analysis of isolated CPMs.

A total of 124 pure strains of pigmented microorganisms were isolated and purified from the enrichment cultures (Table S1). Based on 16S rRNA gene sequence analysis, these strains belong to four phyla (Figure 2A,B): Actinomycetota (31 strains, 25.0%), Bacillota (50 strains, 40.3%), Bacteroidota (3 strains, 2.4%), and Pseudomonadota (40 strains, 32.3%). At the class level, Bacilli (50 strains), Actinomycetia (31 strains), and Alphaproteobacteria (30 strains) were the dominant groups, while Gammaproteobacteria (10 strains) and Flavobacteriia (3 strains) had lower abundances. All strains were classified into eight families. Among them, Planococcaceae (39 strains), Micrococcaceae (31 strains), and Rhodobacteraceae (19 strains) were the three most abundant families, accounting for 71.8% of the total strains. Flavobacteriaceae (3 strains) and Aurantimonadaceae (2 strains) had the lowest abundances, accounting for only 2.4% and 1.6%, respectively. At the genus level, the isolates were distributed among 11 genera. Planococcus (39 strains), Kocuria (25 strains), Paracoccus (19 strains), and Exiguobacterium (11 strains) were predominant, together accounting for 75.8% (94 strains) of the collection. In contrast, genera such as Arthrobacter (3 strains), Citricoccus (3 strains), Aurantimonas (2 strains), Flavobacterium (2 strains), and Olleya (1 strain) included only 11 strains, collectively accounting for 8.9%.

**Figure 3:**
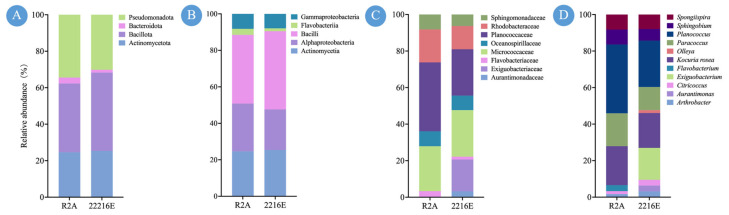
Diversity of CPMs in R2A and 2216E media at phylum (**A**), class (**B**), family (**C**) and genus (**D**) level.

Additionally, the isolation efficiency of R2A and 2216E media for pigmented microorganisms was compared. A total of 61 strains were isolated using R2A medium, while 63 strains were obtained with 2216E medium. At the phylum and class levels, no notable differences were observed in either the number or the taxonomic composition of strains isolated from the two media (Figure 3A,B). At the family level, Exiguobacteriaceae and Aurantimonadaceae were exclusively found in the 2216E medium, whereas Planococcaceae and Rhodobacteraceae showed higher abundance in the R2A medium (Figure 3C). At the genus level, the distribution of strains differed more significantly between the two media. Genera such as Exiguobacterium, Aurantimonas, and Olleya were only isolated from the 2216E medium, while Flavobacterium appeared exclusively in the R2A medium. Furthermore, Planococcus and Paracoccus were more abundant in R2A than in 2216E, whereas no significant differences were observed in the number of strains isolated for genera such as Kocuria, Spongiispira, and Sphingobium between the two media (Figure 3D).

### 2.3. The Influence of Water Depth on the Diversity of CPMs

**Figure 4:**
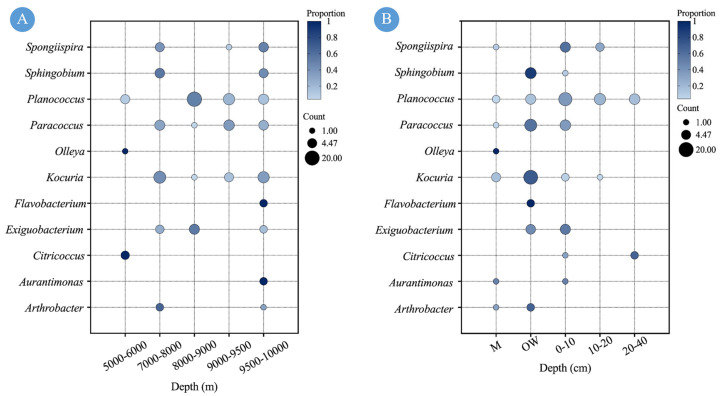
Diversity of CPMs at different water depths (**A**) and in different types of samples (**B**).

Water depth exerted a significant influence on the diversity of carotenoid-producing microorganisms (Figure 4A). The most abundant genus, Planococcus, was detected at all sampled depths from 5951 m to 9588 m, with the exception of the 7305 m station. The highest number of its isolates (20 strains, representing 51.3% of this genus) came from the 8000–9000 m depth interval. In contrast, Kocuria was isolated exclusively from stations deeper than 7000 m, with a majority of strains recovered from the 7000–8000 m (11 strains, 44%) and 9500–10,000 m (9 strains, 36%) ranges. Paracoccus exhibited a similar distribution pattern to Kocuria. Both Exiguobacterium and Spongiispira were found across three depth ranges. However, Exiguobacterium was more abundant within 8000–9000 m, whereas Spongiispira showed higher numbers at 7000–8000 m and 9500–10,000 m. Sphingobium was present in two depth intervals (7000–8000 m and 9500–10,000 m), with no notable difference in strain counts between them. Notably, Citricoccus and Olleya were isolated only from the 5951 m station, while Flavobacterium and Aurantimonas were detected solely in samples from depths exceeding 9500 m.

In addition to sediment cores, water samples were also analyzed. These included the overlying water (OW) above the core surfaces and near-bottom seawater collected as filter samples (M) via an in situ filtration system (0.22 μm) deployed on the submersible. As shown in Figure 4B, a combined total of 63 strains (approximately half of all isolates), representing 10 genera, were isolated from these water samples. The majority (52 strains, 41.9%) originated from OW, whereas only 11 strains (8.9%) came from filter samples (M), underscoring the sediment–water interface as a hotspot for cultivable CPMs. Both diversity and abundance declined markedly with sediment depth. The 0–10 cm layer yielded the highest number of isolates (39 strains, 31.5%, from 8 genera). This number dropped sharply to 13 strains (10.5%, 3 genera) in the 10–20 cm layer and to merely 9 strains (7.3%, 2 genera) in the 20–40 cm layer. Furthermore, the isolated genera displayed distinct ecological preferences. Planococcus, while present in both habitats, showed a clear benthic association, with 31 of its 39 isolates coming from sediment. In contrast, Kocuria was predominantly isolated from water samples (22 of 25 strains), indicating a strong aquatic preference—a pattern shared by Sphingobium and Paracoccus. Notably, niche partitioning was evident even among less abundant genera: Olleya, Flavobacterium, and Arthrobacter were exclusive to water samples, while Citricoccus was found only in sediment.

### 2.4. Carotenoid Characteristics from KKT and Their Biosynthetic Pathways

**Figure 5:**
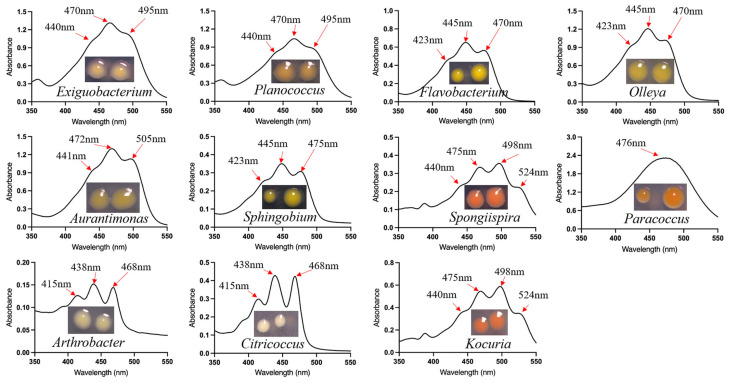
Colony morphology and spectral analysis of carotenoids from KKT-derived CPMs.

CPMs isolated from the KKT exhibited colony pigmentation ranging from light yellow to deep red (Figure 5). To characterize these pigments, we extracted and analyzed the absorption spectra of representative strains from different genera. A clear correlation was observed between pigment spectral profiles and phylogenetic lineage. Strains from the genera Exiguobacterium and Planococcus (phylum Bacillota) formed pale-yellow colonies, with pigment extracts showing absorption maxima at 440 nm, 479 nm, and 495 nm. Similarly, the bacteroidotal genera Flavobacterium and Olleya produced yellow pigments with peaks at 423 nm, 445 nm, and 470 nm. Within the Actinomycetota, genera Arthrobacter and Citricoccus yielded pigments with nearly identical spectra, exhibiting absorption peaks at 415 nm, 438 nm, and 468 nm. Notably, the greatest pigment diversity was found among members of the Pseudomonadota. Within this phylum: Aurantimonas synthesized a light-yellow pigment (maxima at 441 nm, 472 nm, and 505 nm); Sphingobium produced a deep-yellow pigment (423 nm, 445 nm, and 475 nm); Paracoccus yielded a red pigment characterized by a broad absorption band peaking at 476 nm; and Spongiispira generated a deep-red pigment with maxima at 475 nm and 498 nm. Interestingly, phylogenetically distant taxa could produce pigments with similar spectral features. For instance, the deep-red pigments from the actinobacterial genus Kocuria and the pseudomonadotal genus Spongiispira shared identical absorption maxima.

**Figure 6:**
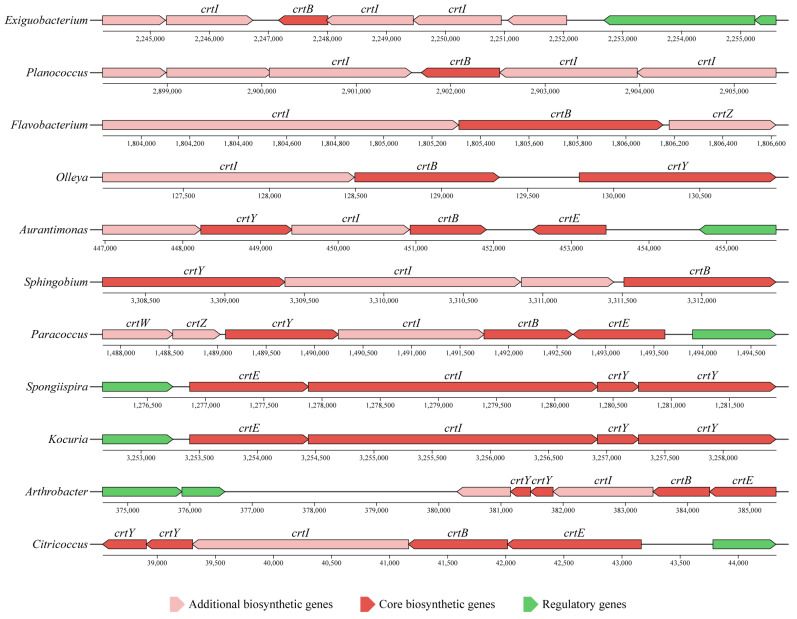
Predicted carotenoid biosynthetic gene clusters based on whole-genome analysis.

The genomes of these carotenoid-producing strains were sequenced, and carotenoid biosynthetic gene clusters (BGCs) were identified using antiSMASH [26]. All 11 genera harbored carotenoid BGCs, and their gene composition and organization correlated with phylogenetic lineage (Figure 6). Within the Bacillota, Exiguobacterium and Planococcus shared an identical set of carotenoid genes (*crtI*, *crtB*, *crtI*, *crtI*) with similar genomic organization. The two Bacteroidota genera possessed conserved *crtI* and *crtB* genes, but differed in additional genes: Flavobacterium contained a *crtZ* (encoding a carotenoid hydroxylase), whereas Olleya carried a *crtY* (encoding a lycopene cyclase). Among Actinomycetota, most genera harbored a common gene set (*crtE*, *crtB*, *crtI*, *crtY*, *crtY*), except for Kocuria, which lacked *crtB* (encoding a phytoene synthase). Considerable variation in BGC composition was observed within the Pseudomonadota. Aurantimonas and Paracoccus shared a core set of genes (*crtY*, *crtI*, *crtB*, *crtE*), but Paracoccus additionally encoded *crtW* and *crtZ* (involved in ketolation and hydroxylation, respectively). Sphingobium contained only *crtY*, *crtI*, and *crtE*, with no predicted *crtB*. Strikingly, the BGC of Spongiispira (*crtE*, *crtI*, *crtY*, *crtY*) closely mirrored that of Kocuria in both composition and organization, despite their phylogenetic distance. Furthermore, these BGCs contained several genes encoding putative modifying enzymes—such as glycosyltransferases, methyltransferases, and acyltransferases—which may participate in carotenoid derivatization and potentially lead to structurally novel carotenoid analogs.

### 2.5. Antioxidant Capacity Analysis of Carotenoid Extracts from KKT Isolates

**Figure 7:**
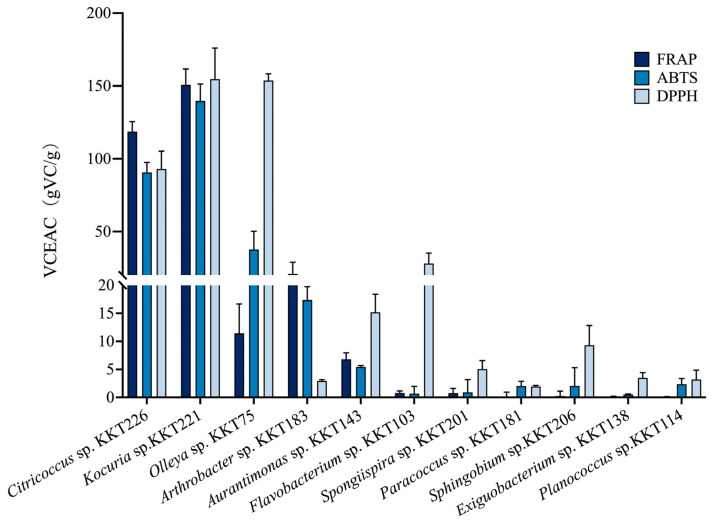
Antioxidant capacity analysis of carotenoid extracts from KKT isolates by FRAP, ABTS, and DPPH Assays.

The antioxidant capacity of carotenoid extracts from KKT isolates was initially evaluated using the ferric reducing antioxidant power (FRAP) assay. As shown in Figure 7, extracts from three actinobacterial genera exhibited relatively strong reducing power. Among these, the extract from *Kocuria* sp. KKT221 showed the highest activity (150.9 ± 10.7 VCEAC), followed by that from *Citricoccus* sp. KKT226 (118.7 ± 6.9 VCEAC). The extract from *Arthrobacter* sp. KKT183 demonstrated lower activity (21.7 ± 7.5 VCEAC). Moderate FRAP activity was observed for extract from *Olleya* sp. KKT75 (12.0 ± 4.7 VCEAC) and *Aurantimonas* sp. KKT143 (6.9 ± 1.0 VCEAC). In contrast, extract from the remaining strains exhibited only weak reducing power.

The free radical scavenging ability of the carotenoid extracts was further assessed via ABTS and DPPH assays. Extracts from Citricoccus, Kocuria, and Olleya showed substantial scavenging capacity in both assays, with ABTS values ranging from 38.8 ± 11.0 to 140.0 ± 11.1 VCEAC and DPPH values from 93.4 ± 11.5 to 155.5 ± 20.0 VCEAC. A distinct pattern was observed for *Arthrobacter* sp. KKT183. Its carotenoids were effective against ABTS radicals (17.5 ± 2.3 VCEAC) but showed negligible activity against DPPH radicals (3.0 ± 0.2 VCEAC). Conversely, extracts from Aurantimonas, Flavobacterium, and Sphingobium exhibited stronger scavenging activity toward DPPH than ABTS radicals. Finally, carotenoids from Spongiispira, Paracoccus, Exiguobacterium, and Planococcus displayed only minimal or negligible activity in both assays.

## 3. Discussion

### 3.1. The Hadal Zone Serves as a Promising Reservoir for Isolating CPMs

Previous studies have shown that HHP elevates intracellular ROS levels [27,28]. Excessive ROS damages critical biomolecules, including proteins, nucleic acids, and lipids, resulting in loss of protein function, DNA lesions, and membrane lipid peroxidation, which can ultimately lead to cellular impairment or death [29,30]. Concurrently, HHP reduces membrane fluidity [31], thereby disrupting essential cellular functions and associated biochemical pathways. Carotenoids, a class of lipid-soluble pigments, possess extended polyene chains with conjugated double-bond systems [32]. This structural feature enables them to effectively quench singlet oxygen, terminate free-radical chain reactions, and inhibit lipid peroxidation, thereby conferring potent antioxidant and free radical-scavenging activities that protect cells from oxidative damage [33,34,35].

We therefore hypothesize that CPMs possess a natural adaptive advantage in hadal environments due to the protective functions of these pigments. Our findings strongly support this hypothesis. First, carotenogenic microorganisms were detected in approximately one-third of the environmental samples from the KKT, indicating their broad distribution in the hadal zone. Second, we successfully isolated 124 carotenoid-producing strains spanning 11 distinct genera, highlighting the considerable phylogenetic diversity of this functional group within the hadal ecosystem. In addition, we cultured these carotenoid-producing microorganisms under different pressure conditions to analyze their pressure tolerance. The results showed that although most strains grew better at atmospheric pressure, they were still able to tolerate up to 100 MPa, suggesting that carotenoid production may help microorganisms survive in the extreme hadal environment characterized by high pressure and low temperature.

Studies on culturable microorganisms from different depths of the Mariana Trench (MT) have also revealed an abundance of CPMs. Zhao et al. isolated 825 culturable bacterial strains belonging to 108 genera from seawater samples collected at various depths of the MT [7]. We analyzed the carotenoid-producing potential of these genera and found that 14 of them harbor carotenoid BGCs in their genomes, suggesting their potential to synthesize carotenoids. These genera comprised 190 strains, accounting for approximately 23% of the total isolates. Among them, the most abundant genera were Erythrobacter, Paracoccus, and Halomonas, which contrasted sharply with the diversity of CPMs isolated from KKT, indicating a geographic specificity of CPMs in different trenches. Notably, among the CPMs in the MT, 113 strains were derived from samples collected at depths exceeding 6000 m, representing about 60% of the total CPMs. In contrast, approximately half of the non-pigmented microorganisms originated from samples deeper than 6000 m. These findings further suggest that the extreme hadal environment favors the enrichment of CPMs.

### 3.2. The Importance of Cultivation Strategies for Recovering Microbial Diversity

The distinct physicochemical conditions of the hadal zone shape unique microbial communities and metabolic traits, which differ markedly from those inhabiting surface or shallow marine environments [36]. Studies on Mariana Trench sediments further suggest that microbial novelty increases with depth [9]. To maximize the recovery of carotenoid-producing microbial diversity from hadal samples, we employed two nutrient-diverse media, R2A and 2216E, for enrichment cultivation. This strategy yielded 124 isolates in total, with comparable numbers recovered from R2A (61 isolates) and 2216E (63 isolates).

Despite the similar total yields, the taxonomic composition of isolates from the two media differed substantially. R2A medium supported the growth of isolates from 7 genera, predominantly Planococcus (18.5% of total isolates), Kocuria (10.5%), and Paracoccus (8.9%). In contrast, 2216E medium yielded a more diverse assemblage spanning 10 genera, with dominant taxa including Planococcus (12.9%), Kocuria (9.7%), Exiguobacterium (8.9%), and Paracoccus (6.5%). Notably, several genera showed medium specificity: Exiguobacterium, Aurantimonas, and Olleya were isolated exclusively on 2216E, while Flavobacterium was recovered only on R2A. The relative abundance of shared taxa also differed between the two media. For instance, Planococcus and Paracoccus were proportionally more abundant in R2A. In addition, we analyzed the isolation of potential novel species from the two media. Using a 16S rRNA gene sequence similarity threshold of <98.65% for preliminary novel species identification, a total of 18 potential novel species were obtained. Among these, 14 belonged to the genus Paracoccus (9 isolated from R2A medium and 5 from 2216E medium), and 4 belonged to the genus Planococcus (1 from R2A medium and 3 from 2216E medium). Furthermore, we performed genome sequencing on selected strains and found that although some strains exhibited >99% 16S rRNA gene similarity, three strains had digital DNA–DNA hybridization (dDDH) values below 70% with their type strains, indicating that they represent potential novel species. Collectively, these results demonstrate that the use of multiple cultivation media is crucial for maximizing the taxonomic recovery of CPMs from hadal environments.

Furthermore, while this study examines the influence of culture medium, sample depth, and location on the diversity and abundance of CPMs, we emphasize that culture-dependent diversity analysis is limited to the conditions applied and does not accurately capture the native microbial community structure in situ.

### 3.3. The Carotenoid Biosynthesis Potential of Microorganisms Derived from the KKT

In this study, the assignment of carotenoids based solely on UV–Vis spectra remains preliminary; however, genome mining provided complementary insights that significantly strengthened our analysis. For instance, strains isolated from the genera Exiguobacterium and Planococcus exhibited UV–Vis absorption spectra with three characteristic peaks (440 nm, 479 nm, and 495 nm) and harbored BGCs homologous to *crtB* and *crtI*. The architecture of these gene clusters (*crtI*-*crtB*-*crtI*-*crtI*) shows high similarity to the established C30 carotenoid biosynthesis pathways identified in Exiguobacterium [37] and Planococcus [38] species. Strains of the genera Flavobacterium and Olleya displayed similar UV–Vis absorption spectra (423 nm, 445 nm, and 470 nm) but differed slightly in their carotenoid BGCs: Flavobacterium possessed a single copy of *crtZ*, whereas Olleya contained a single copy of *crtY*. Previous studies have shown that most Flavobacterium strains harbor a conserved *crtI-B-Z* gene cluster along with *crtY* and are capable of synthesizing zeaxanthin [39]. Given that the absorption spectra of the extracts from both genera in this study are similar to those of known zeaxanthin-producing strains, we hypothesize that they may also possess the ability to produce zeaxanthin. Within the phylum Actinomycetota, strains of the genera Arthrobacter and Citricoccus shared identical UV–Vis absorption spectra (415 nm, 438 nm, and 468 nm) and similar gene cluster compositions (*crtE-crtB-crtI-crtY-crtY*). The carotenoid BGCs in Arthrobacter are relatively conserved and are known to direct the synthesis of rare C_50_ carotenoids [40,41]. Based on this, we infer that strains of Arthrobacter and Citricoccus also have the potential to produce C_50_ carotenoids. Furthermore, the genus Kocuria (phylum Actinomycetota) and the genus Spongiispira (phylum Pseudomonadota) exhibited similar UV–Vis absorption spectra for carotenoids (440 nm, 475 nm, 498 nm, and 524 nm) and shared similar BGCs (*crtE-crtI-crtY-crtY*). Research has demonstrated that *Kocuria* sp. RAM1 produces a red C_50_ carotenoid with absorption peaks at 475 nm, 500 nm, and 535 nm [42]. Consequently, we propose that the Kocuria and Spongiispira strains isolated from the KKT also possess the potential for C_50_ carotenoid biosynthesis. Within the phylum Pseudomonadota, strains of genera such as Aurantimonas, Sphingobium, and Paracoccus displayed differences in both their UV–Vis absorption spectra and gene cluster compositions, suggesting they may synthesize different types of carotenoids. Indeed, Paracoccus species are known for their high capacity to produce astaxanthin [43], and Sphingobium species can synthesize zeaxanthin [44]. For the Aurantimonas strains, although reports on the identification of their specific carotenoids are scarce, they share similar gene clusters (*crtY-crtI-crtB*) with Sphingobium, indicating a potential for β-carotene biosynthesis [45]. This integrative analysis, linking genotype with spectral characteristics, lays the foundation for more targeted identification of carotenoids in future studies employing mass spectrometry.

### 3.4. Antioxidant Activity of Carotenoids Derived from Hadal Microorganisms

The extreme hadal environment is hypothesized to select for uniquely modified carotenoids (e.g., via glycosylation, or distinct stereochemistry), which may possess novel bioactivities [35,46]. To explore this functional potential, we systematically evaluated the antioxidant capacity of carotenoid extracts from KKT isolates using FRAP, ABTS, and DPPH assays (Figure 7). A clear phylogenetic correlation was observed: closely related strains exhibited similar antioxidant profiles. Specifically, Actinobacterial genera (Citricoccus, Kocuria, and Arthrobacter) displayed strong overall antioxidant capacity. Bacteroidotal strains (Olleya and Flavobacterium) showed pronounced activity against DPPH radicals, whereas Bacillotal representatives (Exiguobacterium and Planococcus) were relatively weak. Interestingly, carotenoid extracts with similar spectral profiles could differ markedly in function. For instance, extracts from Kocuria and Spongiispira, both showing absorption peaks near 440, 475, 498, and 524 nm, could differ markedly in antioxidant potency. Furthermore, genus-specific scavenging preferences were evident: Citricoccus and Kocuria neutralized ABTS and DPPH radicals with comparable efficiency; Arthrobacter preferentially quenched ABTS radicals; and Aurantimonas and Flavobacterium were more effective against DPPH. Together, carotenoids from the hadal zone exhibit considerable structural and functional diversity. Their antioxidant properties appear to be governed by a complex interplay between specific chemical structures and phylogenetic lineage.

## 4. Materials and Methods

### 4.1. Sample Collection and Bacterial Isolation

During the TS42-1 expedition conducted between June and July 2024, sediment samples were obtained along the axial zone of the Kuril–Kamchatka Trench using the manned submersible “Fendouzhe”. Sampling was performed at 15 stations across a depth gradient spanning 5951 to 9588 m. Concurrently, during near-bottom operations, approximately 200 L of seawater was filtered in situ through 0.22 μm pore-size membranes via a filtration unit mounted at the submersible’s stern.

Once the samples were retrieved on deck, they were immediately processed in a cold room, and microbial enrichment cultures were initiated in the shipboard laboratory. The entire procedure was completed within a few hours to avoid the effects of sample storage on the abundance and diversity of culturable microorganisms. For sediment cores, overlying water was collected from the sediment surface, and the remaining sediment was sectioned at 2 cm intervals. Aliquots (50 μL) of the overlying water were spread onto both 2216E and R2A agar plates. Approximately 0.5 g of sediment from each interval was suspended in 5 mL of sterile seawater, and 50 μL of the suspension was plated onto the same two media types. Filter samples were processed by cutting one-quarter of each membrane, which was then resuspended in 1 mL of sterile seawater with vortex mixing. A 50 μL aliquot of this suspension was plated onto 2216E and R2A media. All inoculated plates were incubated in the dark at 4 °C for approximately one month. Pigmented colonies were then picked and isolated by streaking onto fresh 2216E or R2A agar. This purification procedure was repeated three times, and culture purity was assessed based on colony morphology and 16S rRNA gene sequencing. Purified strains were cultivated in appropriate liquid medium, preserved with 10% (*v*/*v*) DMSO as a cryoprotectant, and stored at −80 °C for long-term maintenance.

### 4.2. 16S rRNA Gene PCR Amplification and Sequencing Analysis

A single colony was picked from the plate and suspended in 500 µL of sterile water. After vortex mixing, the cells were collected by centrifugation at 12,000× *g* for 5 min. The pellet was resuspended in 100 µL of sterile water, heated at 100 °C in a metal bath for 10 min, and centrifuged again at 12,000× *g* for 5 min. The resulting supernatant was collected and used as the DNA template. The bacterial 16S rRNA gene was amplified using primers 27F (5′-AGAGTTTGATCCTGGCTCA-3′) and 1492R (5′-GGTTSCCTTGTTACGACTT-3′). PCR was performed in a 25 µL reaction mixture containing 12.5 µL of Premix Taq mix, 1 µL of each primer (20 µmol/L), and 1 µL of genomic DNA. The thermal cycling conditions were: initial denaturation at 98 °C for 30 s; 30 cycles of denaturation at 98 °C for 10 s, annealing at 55 °C for 30 s, and extension at 72 °C for 1.5 min; followed by a final extension at 72 °C for 10 min, and hold at 4 °C. PCR products were verified by 1% agarose gel electrophoresis and then sent to RuiBiotech (Beijing, China) for 16S rRNA gene sequencing. The obtained sequences were subjected to BLAST analysis against the NCBI GenBank database to determine the taxonomic affiliation of the isolates.

### 4.3. Extraction and Quantification of Carotenoids

Carotenoid-producing strains were inoculated into 200 mL of 2216E or R2A liquid medium and cultured at 4 °C with shaking (150 rpm) for approximately one week. Growth was monitored by measuring the optical density at 600 nm (OD600) to determine biomass. After reaching the stationary phase, cells were harvested by centrifugation (10,000× *g*, 10 min, 4 °C). The cell pellet was resuspended in an appropriate volume of methanol and vortex-mixed. The centrifuge tubes were wrapped in aluminum foil to protect them from light and placed on a horizontal shaker for extraction until the cells were fully bleached. Following extraction, the mixture was centrifuged at 10,000× *g* for 10 min, and the supernatant was collected as the crude pigment extract. The absorption spectrum of the crude extract (200–1100 nm) was recorded using a Cary 60 UV–Vis spectrophotometer (Agilent Technologies, Santa Clara, CA, USA), with methanol as the blank. The total carotenoid (mg/L culture liquid) was quantified using the following formula [47]:

$$\frac{A\times D\;\times \;10^{4}}{E_{1\;\mathrm{cm}}^{1\%}}$$

where A = absorbance of diluted extract solution; D = the dilution ratio; $E_{1\;cm}^{1\%}$ is the extinction coefficient of carotenoid. 2660, absorption coefficient of C_50_ carotenoids in methanol [47], which was used to calculate the carotenoid content of the genera Arthrobacter, Citricoccus, Kocuria and Spongiispira; 2592, absorption coefficient of β-carotene in hexane [48], which was used to calculate the carotenoid content of the genus Aurantimonas; 2540, absorption coefficient of zeaxanthin in ethanol [48], which was used to calculate the carotenoid content of the genera Flavobacterium, Olleya and Sphingobium; 2500 was used to calculate the carotenoid content of the genera Exiguobacterium and Planococcus [49]; and 2100 was used to calculate the carotenoid content of the genus Paracoccus [50].

### 4.4. Determination of the Antioxidant Activities of Carotenoids

The total antioxidant capacity of carotenoid crude extracts was evaluated using three complementary assays: ferric reducing antioxidant power (FRAP), and ABTS and DPPH radical scavenging activities. For the FRAP assay, the reagent was prepared according to the manufacturer’s instructions (Total Antioxidant Capacity Assay Kit with FRAP method, S0116, Beyotime Institute of Biotechnology, Shanghai, China) two hours before use and pre-warmed to 37 °C in a water bath. A 5 μL aliquot of each diluted sample was mixed with 180 μL of the FRAP reagent. After a 10-min incubation, absorbance was measured at 593 nm using a Varioskan LUX microplate reader (Thermo Fisher Scientific, Waltham, MA, USA). A standard curve was constructed using vitamin C (VC) as a reference, with VC standard solutions prepared at concentrations of 0.3, 0.25, 0.125, 0.0625, 0.03125, 0.015625, 0.0078125, and 0.00390625 mg/mL. The antioxidant capacity was calculated based on the standard curve and expressed as vitamin C equivalent antioxidant capacity (VCEAC). Similarly, the DPPH radical scavenging activity was determined using the DPPH Free Radical Scavenging Capacity Assay Kit (BC 4750, Solarbio, Beijing, China), and the ABTS radical scavenging activity was assessed using the Total Antioxidant Capacity Assay Kit with Rapid ABTS method (S0121, Beyotime Institute of Biotechnology, Shanghai, China).

### 4.5. Genome Sequencing and Analysis of Carotenoid Biosynthesis Gene Clusters

The carotenoid-producing microorganism was inoculated into 2216E medium and cultured with shaking at 150 rpm at 4 °C until the end of the logarithmic growth phase (OD600 value of 0.7–0.8). The cells were harvested by centrifugation at 4500× *g* for 30 min at 4 °C. Genomic DNA was extracted according to the instructions of the MagAttract DNA Kit (Hilden, Germany), and its purity, concentration, and integrity were assessed using Nanodrop, Qubit, and 0.35% agarose gel electrophoresis. Large DNA fragments were recovered using the BluePippin automated nucleic acid recovery system, and a library was constructed using the Oxford Nanopore ligation kit (SQK-LSK109, Hangzhou, China). Subsequently, sequencing was performed using the BGI SEQ-500 platform and the Oxford Nanopore Technologies (ONT) platform. After filtering out low-quality and short reads, the filtered reads were assembled using Canu v1.5 software (https://github.com/marbl/canu, accessed on 23 June 2025), and further error correction was performed using Pilon v1.22 software (https://github.com/broadinstitute/pilon, accessed on 26 June 2025) in conjunction with second-generation sequencing data. The completeness and quality of the bacterial genome assembly were evaluated using BUSCO v5.8.2 and CheckM v1.1.3.

Gene prediction for the assembled genome was conducted using Prodigal v2.6.3 (https://github.com/hyattpd/Prodigal, accessed on 30 June 2025), and the prediction of secondary metabolite biosynthetic gene clusters in the strain’s genomic sequence was performed using the antiSMASH online server (version 8.0, https://antismash.secondarymetabolites.org/#!/start, accessed on 13 December 2025) with the detection strictness set to “relaxed” mode, obtaining the location information and functional annotations of the gene clusters. The annotated gene cluster information was then compiled and uploaded to the ChiPlot online platform (https://www.chiplot.online/, accessed on 26 December 2025) for visualization, generating a gene cluster structure map.

### 4.6. Nucleotide Sequence Accession Numbers

The 16S rRNA gene sequences of 124 CPMs were deposited in the GenBank (NCBI) database with the accession numbers PX312534-PX312687. The accession numbers of the genomes used in this study are: JBVMVX000000000, JBVOUJ000000000, JBVMVY000000000, JBVMWA000000000, JAAAMM000000000, JBVMWC000000000, JBVMWB000000000, JBVMVZ000000000, JBVMWD000000000, JBVMVW000000000, and JBHLWH000000000.

## 5. Conclusions

This study demonstrates that the extreme hadal environment (characterized by high pressure and low temperature) selectively enriches for CPMs, establishing it as a prime source for this functional group. By employing media with different nutrient profiles, we significantly enhanced both the recovery and phylogenetic diversity of these ecologically adapted strains. Furthermore, the derived carotenoids exhibited potent antioxidant and free radical scavenging capacities. Notably, the biosynthetic pathways and specific antioxidant profiles of these pigments showed a strong correlation with the phylogenetic lineage of the producing strains. In conclusion, our work reveals the hadal zone as a significant and largely untapped reservoir of phylogenetically diverse carotenogenic microorganisms. These findings not only provide a foundational resource for the discovery of novel carotenoids with unique bioactivities but also highlight the potential of extreme-environment microbes for biotechnological applications.

## Data Availability

The data presented in this study are available in the article and its Supplementary Information.

## Supplementary Materials

The following supporting information can be downloaded at: https://www.mdpi.com/article/10.3390/md24030105/s1, Table S1: Information of all the carotenoid-producing microorganisms from the Kuril-Kamchatka Trench.
